# Deep learning model based on ultrasound images predicts BRAF V600E mutation in papillary thyroid carcinoma

**DOI:** 10.1016/j.isci.2025.112482

**Published:** 2025-04-18

**Authors:** Yiwen Yu, Chengqian Zhao, Ruohan Guo, Yafang Zhang, Xiaoxian Li, Naxiang Liu, Yun Lu, Xu Han, Xiaofeng Tang, Rushuang Mao, Chuan Peng, Jinhua Yu, Jianhua Zhou

**Affiliations:** 1 Department of Ultrasound, Sun Yat-Sen University Cancer Center, State Key Laboratory of Oncology in South China, Collaborative Innovation Center for Cancer Medicine, Guangzhou, China; 2 School of Information Science and Technology, Fudan University, Shanghai, China; 3 Department of Ultrasonography, Clinical Oncology School of Fujian Medical University, Fujian Cancer Hospital, Fujian Branch of Fudan University Shanghai Cancer Center, Fuzhou, China; 4 Department of Functional, Sun Yat-sen University Cancer Center Gansu Hospital, Gansu Provincial Cancer Hospital, Lanzhou, China

**Keywords:** cancer, computer modeling, in silico biology

## Abstract

BRAF V600E mutation status detection facilitates prognosis prediction in papillary thyroid carcinoma (PTC). We developed a deep-learning model to determine the BRAF V600E status in PTC. PTC from three centers were collected as the training set (1341 patients), validation set (148 patients), and external test set (135 patients). After testing the performance of the ResNeSt-50, Vision Transformer, and Swin Transformer V2 (SwinT) models, SwinT was chosen as the optimal backbone. An integrated BrafSwinT model was developed by combining the backbone with a radiomics feature branch and a clinical parameter branch. BrafSwinT demonstrated an AUC of 0.869 in the external test set, outperforming the original SwinT, Vision Transformer, and ResNeSt-50 models (AUC: 0.782–0.824; *p* value: 0.017–0.041). BrafSwinT showed promising results in determining BRAF V600E mutation status in PTC based on routinely acquired ultrasound images and basic clinical information, thus facilitating risk stratification.

## Highlights

• BrafSwinT is an AI model that predicts BRAF V600E mutation status in PTC
• BrafSwinT includes a deep-learning, a radiomics, and a clinical parameter branch
• BrafSwinT surpassed ResNeSt-50, Vision Transformer, and Swin Transformer V2 models

## Graphical abstract

**Figure:**
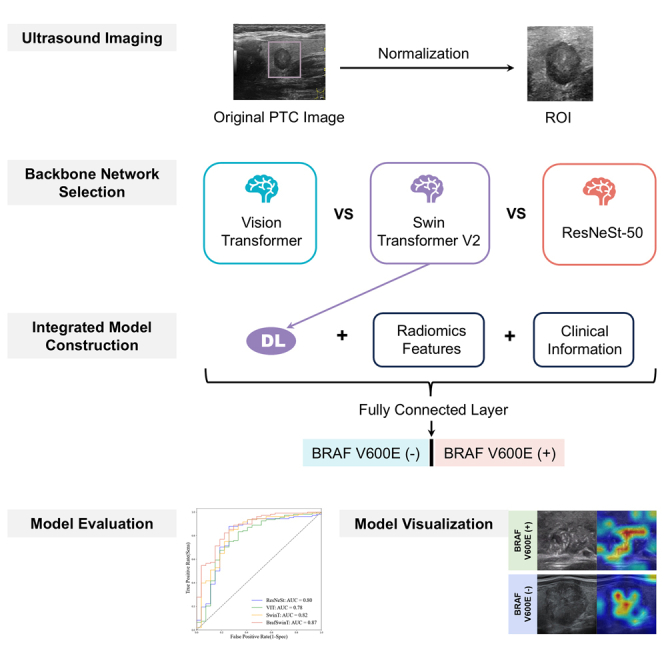

## Introduction

Due to the rapid development of high-resolution ultrasound technology and the health consciousness of the whole population, the reported global incidence of thyroid cancer nearly tripled from 1990 to 2019.^1^ For higher-risk thyroid cancer patients, more vigorous interventions are recommended, such as unilateral thyroid lobectomy or bilateral thyroidectomy, with or without lymph node dissection, radioiodine therapy, and targeted medication. Meanwhile, for some lower-risk patients, close surveillance,^2^ or minimally invasive treatments like thermal ablation^3^ might be preferred. To avoid the misallocation of medical resources, patient risk stratification and individualized management should be carefully considered.

In previous studies, the prevalence of BRAF V600E mutation was 40%–50% in Caucasian papillary thyroid carcinoma (PTC) patients,^4^ and ranged from 72%–81% in Asian PTC patients.^5^^,^^6^ PTC patients with BRAF V600E mutation are more vulnerable to disease recurrence and less sensitive to radioactive iodine therapy compared to those without the mutation.^7^^,^^8^^,^^9^^,^^10^ BRAF inhibitors may be a possible therapy for patients suffering from repeated recurrence or distant metastasis.^11^ Furthermore, PTCs with BRAF V600E mutation are more prone to lymph node metastasis,^12^ even in clinically node-negative patients,^13^ which poses a relative contradiction for active surveillance or minimally invasive treatments. Therefore, detecting the BRAF V600E mutation can help with treatment decisions and prognosis prediction in patients with PTC.

Currently, BRAF V600E mutation detection is conducted using surgical or fine needle aspiration specimens, relying on Sanger sequencing, amplification refractory mutation system-quantitative polymerase chain reaction, or next-generation sequencing technology. The methods aforementioned consume a substantial amount of time and money. In addition, the accuracy of the diagnosis based on fine needle aspiration specimens relies on the technique of the operators as well as the homogeneity of the lesions. Therefore, there is a strong need to develop a simple and noninvasive method to detect the BRAF V600E mutation.

Radiomics features extracted from clinical imaging were found to be associated with specific gene expression patterns or molecular phenotypes, leading to the rise of radiogenomics.^14^ For instance, Meissner and colleagues^15^ established a radiomics model based on magnetic resonance imaging to predict the BRAF mutation in melanoma brain metastases with an area under the receiver operating characteristic curve (AUC) of 0.92, suggesting the significant association between BRAF mutation and radiomics features. Recently, radiomics models have been developed using grayscale US images to predict the BRAF V600E mutation in PTC patients. However, unsatisfying results were reported,^16^^,^^17^^,^^18^^,^^19^ with the possible causes of small sample sizes and suboptimal modeling methods.

Deep learning models can automatically extract features from input images, constructing a hierarchy of concepts to learn complicated patterns by building them from simpler ones. This process simulates the neural network of human brain more effectively than other machine learning methods. Among the thriving deep learning methods for natural language processing, several models stood out in handling medical image segmentation tasks. The Swin Transformer model V2 (SwinT)^20^ incorporated a shifted window-attention mechanism that enhances local perceptibility, allowing the model to concurrently extract multi-scale global and local features, thereby fostering comprehensive feature representation. The ResNeSt-50 (ResNeSt) model integrated Convolutional Neural Networks with a split-attention mechanism, enhancing its capacity to derive richer and more expressive feature representations. Additionally, the Vision Transformer (ViT) model’s global self-attention mechanism proved pivotal in capturing intricate relationships between diverse regions within the image. It was hypothesized that the unrecognized US phenotype related to BRAF V600E mutation might be better identified by these state-of-the-art deep learning models. Furthermore, the original models above were expected to be optimized by modifying and integrating image features with clinical parameters.

The presented study aimed to develop a deep-learning model to determine the BRAF V600E mutation status in PTC. This model utilized a pre-trained network based on US images as its foundational framework and was further enhanced by integrating other radiomics features and basic clinical information.

## Results

### Baseline characteristics

**Figure 1:**
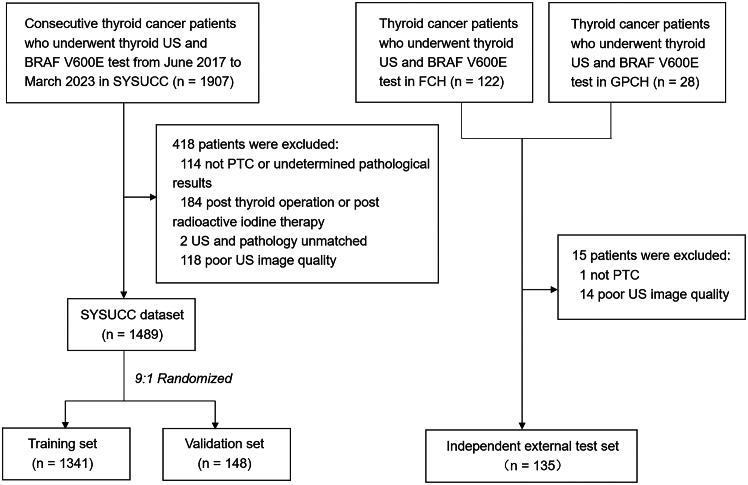
Patient enrollment workflow The patients were retrospectively collected in three cohorts: 1341 PTC patients from SYSUCC as the training cohort, 148 PTC patients from SYSUCC as the validation cohort, and 135 PTC patients from two other centers as the external test cohort. US, ultrasound; SYSUCC, Sun Yat-Sen University Cancer Center; PTC, papillary thyroid carcinoma; FCH, Fujian Cancer Hospital; and GPCH, Gansu Provincial Cancer Hospital.

In this multicenter retrospective study, a total of 1341 PTC patients from SYSUCC were investigated as the training set for model development. Another cohort of 148 PTC patients from SYSUCC served as the internal validation set, while 135 PTC patients from the Gansu Provincial Cancer Hospital and the Fujian Provincial Cancer Hospital were collected as the independent external test set (see Figure 1).

The baseline parameters among the three cohorts are presented in Table 1. Notably, there was no significant difference in the percentage of BRAF V600E mutation among the three cohorts (*p* = 0.460). The nodule size in the training set was larger compared to those in both the validation and external test sets.

**Table 1:** Baseline characteristics of papillary thyroid carcinoma patients in the training set, validation set and external test set

	Training set (*n* = 1341)	Validation set (*n* = 148)	External test set (*n* = 135)	*p* value
Age (y, mean ± SD)	39.12 ± 11.19	35.03 ± 9.02	44.24 ± 11.07	<0.001
Sex (n [%])				0.414
Male	408 (30.4%)	40 (27.0%)	35 (25.9%)	
Female	933 (69.6%)	108 (73.0%)	100 (74.1%)	
Tumor size (mm, median [IQR])	10.00 (7.00–15.00)	8.00 (6.00–12.25)	8.57 (6.53–12.04)	<0.001
BRAF V600E mutation status (n [%])				0.460
Positive	1008 (75.2%)	112 (75.7%)	108 (80.0%)	
Negative	333 (24.8%)	36 (24.3%)	27 (20.0%)	

SD, standard deviation; IQR, inter quartile range.

### The performance of the candidate backbone models

The prediction efficiency of the original SwinT, ViT, and ResNeSt models is reported and compared in Table 2. The SwinT model showed higher prediction efficacy in determining BRAF V600E mutation in PTC using US images with an AUC of 0.877 in the validation set and 0.824 in the external test set, outperforming the ResNeSt and the ViT models (AUC: 0.836 and 0.869 in the validation set, 0.800 and 0.782 in the external test set).

**Table 2:** Performance of BrafSwinT, ResNeSt-50, Vision Transformer, and Swin Transformer V2 model to predict the BRAF V600E mutation status in PTC

Cohort	Model	AUC	ACC	SENS	SPEC	PPV	NPV	*p* value^1^	*p* value^2^
Training set	ResNeSt	0.837	81.7%	84.4%	73.7%	90.7%	61.5%	0.007	0.054
	ViT	0.858	83.4%	86.1%	75.2%	91.3%	64.7%	0.048	0.090
	SwinT	0.914	90.7%	92.1%	86.5%	95.4%	78.9%	NA	0.062
	BrafSwinT	0.952	92.7%	93.6%	90.2%	96.7%	82.9%	NA	NA
Validation set	ResNeSt	0.836	81.8%	83.9%	75.0%	91.3%	60.0%	0.041	0.015
	ViT	0.869	83.8%	86.6%	75.0%	91.5%	64.3%	0.089	0.040
	SwinT	0.877	86.5%	88.4%	80.6%	93.4%	69.0%	NA	0.040
	BrafSwinT	0.911	87.8%	90.2%	80.6%	93.5%	72.5%	NA	NA
External test set	ResNeSt	0.800	78.5%	83.3%	59.3%	89.1%	47.1%	0.071	0.022
	ViT	0.782	81.5%	85.2%	66.7%	91.1%	52.9%	0.043	0.017
	SwinT	0.824	83.7%	88.0%	66.7%	91.3%	58.1%	NA	0.041
	BrafSwinT	0.869	88.1%	90.7%	77.8%	94.2%	67.7%	NA	NA

PTC, papillary thyroid carcinoma; AUC, the area under the receiver operating characteristic curves; ACC, accuracy; SENS, sensitivity; SPEC, specificity; PPV, positive predictive value; NPV, negative predictive value; ResNeSt, ResNeSt-50; ViT, Vision Transformer; SwinT, Swin Transformer V2.

*p* value^1^, calculated by comparing AUC of the model to that of SwinT.

*p* value^2^, calculated by comparing AUC of the model to that of BrafSwinT.

### Integrated model development

Due to the superior performance of the SwinT model in the network selection phase, it was determined to be the backbone of the multi-scale feature branch. The SwinT, pre-trained on the ImageNet dataset, excels at capturing long-range dependencies between pixels in images, enabling it to extract global information both within the nodule and along its boundaries. In this branch, each image was initially divided into non-overlapping patches using a patch partition module. Subsequently, a cascade of N (where *N* = 4) multi-scale feature encoders was employed to encode spatial features. Each encoder consisted of L (where L = 2, 2, 6, 2) Swin Transformer blocks. The resulting four-scale features were used for the classification task.

Inaccuracies in multi-scale feature extraction for classification prediction often arise from image marker contamination. To mitigate this issue, the second branch, namely the radiomics feature branch, was designed inspired by the work of Yu et al.^21^ Initially, a 2D wavelet transform was applied to the region of interest (ROI) images, capturing a 64 × 64 patch at the center to accentuate the nodule’s internal region. A set of 70 radiomics features was extracted from the ROI patches, and then 280 radiomics features were derived from the corresponding wavelet transform images. Additionally, the presence or absence of the measuring markers, which included dots or lines on the US images, was put in as a dichotomous variable. As displayed in Table 3, adding the second branch improved the AUC of SwinT by 0.03, specificity by 7.9%, and negative predictive value (NPV) by 4.9% in the external test set.

**Table 3:** Additional performance gains from the inclusion of radiomic features on the base of Swin Transformer V2, and from clinical information on the base of Swin Transformer V2 concatenated with the radiomic features

Branch of BrafSwinT	Cohort	AUC	ACC	SENS	SPEC	PPV	NPV
+ Radiomic features	Training set	+0.02	+1.2%	+0.8%	+2.0%	+0.6%	+1.5%
	Validation set	+0.01	+0.7%	+1.0%	+0.0%	+0.0%	+1.7%
	External test set	+0.03	+2.5%	+1.0%	+7.9%	+1.8%	+4.9%
+ Clinical information	Training set	+0.02	+0.8%	+0.7%	+1.7%	+0.7%	+2.5%
	Validation set	+0.02	+0.6%	+0.8%	+0.0%	+0.1%	+1.8%
	External test set	+0.02	+1.9%	+1.7%	+3.2%	+1.1%	+4.7%

AUC, the area under the receiver operating characteristic curves; ACC, accuracy; SENS, sensitivity; SPEC, specificity; PPV, positive predictive value; NPV, negative predictive value.

Furthermore, a third branch of clinical parameters was fused into the former two branches. For each ROI image, the radiomics features were concatenated with the corresponding multi-scale features and then integrated with three basic clinical parameters of the patient (sex, age, and nodule size). The basic clinical parameters were represented as a one-dimensional array. For example, a 37-year-old male patient with a PTC nodule size of 11 mm was recorded as (1, 37, and 11). As displayed in Table 3, adding the third branch improved the AUC of the other two branches by 0.02%, specificity by 3.2%, and NPV by 4.7% in the external test set.

**Figure 2:**
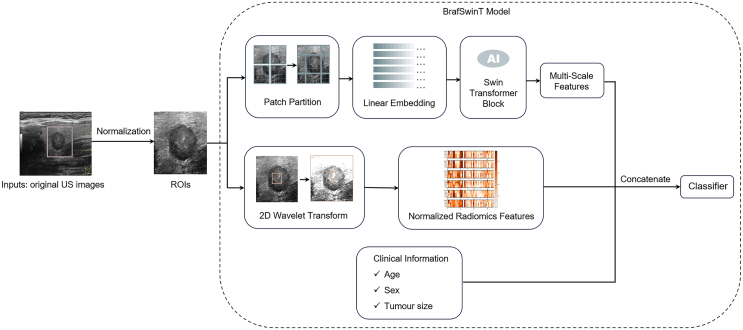
The pipeline of the BrafSwinT model The model contained three parallel branches, wherein multi-scale features, radiomics features, and clinical parameters were fused into a linear classifier. ROI, region of interest.

After the three parallel branches above, a fully connected layer was adopted as the linear classifier to provide the computer-aided diagnosis of positive or negative BRAF V600E mutation status for each image (see Figure 2).

In summary, the integrated model, namely the multi-feature fusion swin transformer model for BRAF V600E diagnosis (BrafSwinT), took US images and clinical parameters as input and produced classification results of each image as output. We apply 5-fold cross-validation to train the BrafSwinT. The samples contained in each fold are non-overlapping and mutually exclusive.

The proposed BrafSwinT was trained for 100 epochs with an SGD optimizer, batch size of 2, and weight decay of 0.05. The initial learning rate was set to 2 × 10−3 and decayed in a cosine schedule with 2.5 epochs of linear warm-uptrained. All experiments were based on Python v3.6 and PyTorch v1.7.1 library, and it ran on AMD EPYV 7532 32-Core Processor @ 2.40 GHz with 256 GB RAM and 3 Nvidia GeForce RTX 3090 with CUDA v11.0 and cuDNN v8.1.0. The trainable parameters of the proposed BrafSwinT are 29.6 M. This allows our model to be easily deployed on medical devices.

### The performance of BrafSwinT and comparison with the SwinT, ViT, and ResNeSt models

The integrated BrafSwinT model showed an AUC of 0.911 in the internal validation set and 0.869 in the external test set (see Table 2). The model demonstrated satisfying positive predictive values of 93.5% in the validation set and 94.2% in the external test set, but less satisfying negative predictive values of 72.5% and 67.7%, respectively.

**Figure 3:**
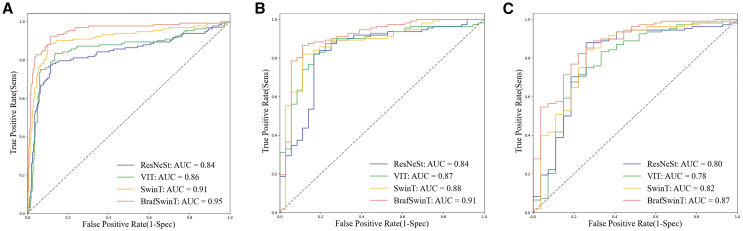
The receiver operating characteristic curves of the deep-learning models Comparison of the receiver operating characteristic curves among different deep-learning models to predict the BRAF V600E mutation status in papillary thyroid carcinoma in (A) the training set, (B) the internal validation set, and (C) the external test set ResNeSt, ResNeSt-50; ViT, Vision Transformer; SwinT, Swin Transformer V2.

The BrafSwinT model showed superior performance in predicting the BRAF V600E mutation status in PTC than the unmodified deep learning models. The AUC of the BrafSwinT model (AUC: 0.869) was significantly higher than that of the SwinT (AUC: 0.824; *p* = 0.041), ViT (AUC: 0.782; *p* = 0.017), and ResNeSt (AUC: 0.800; *p* = 0.022) models in the external test set. The receiver operator characteristic curves of the four models are shown in Figure 3. The BrafSwinT showed a sensitivity of 90.2% in the validation set and 90.7% in the external test, slightly higher than the original SwinT model (sensitivity: 88.4% in the validation set, 88.0% in the external test set). As for specificity, the improvement approach for the construction of BrafSwinT model elevated the value from 66.7% of SwinT to 77.8% in the external test set. The confusion matrix of the performance of BrafSwinT in the external test set was displayed in the Table S1.

### Visual interpretation of the BrafSwinT model

**Figure 4:**
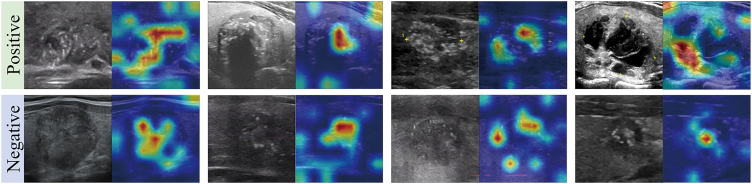
Heat maps of the BrafSwinT model The areas in the ultrasound images that the model assigned the most significance to were the microcalcification zones, as well as the hypoechoic zones that were fully or partially surrounded by hyperechoic zones.

As illustrated in Figure 4, heatmaps were generated to visualize the texture structure features for classification by BrafSwinT with a gradient distribution of colors. The model put the most weight on the microcalcification zones, as well as the hypoechoic zones that were fully or partially surrounded by hyperechoic zones.

## Discussions

This study established an integrated deep learning model, BrafSwinT, to predict the BRAF V600E mutation status in PTC patients based on gray-scale US images of the thyroid nodules and basic clinical data. The BrafSwinT model demonstrated favorable performance in the current task, surpassing several other deep learning models, including the original SwinT, ViT, and ResNeSt. Using routinely acquired US and clinical data, BrafSwinT can facilitate treatment decisions and risk stratification in PTC, while saving time and costs for the patients.

The relationship between thyroid US images and BRAF V600E mutation status in PTC has been explored using multiple methods. Several studies using conventional statistical strategies like logistic regression revealed that certain US features recognized by the radiologists, such as microcalcification and lobulated or irregular margin, were significantly associated with the BRAF V600E mutation status.^22^^,^^23^ These findings suggested the potential for further investigation into radiomics models to predict the BRAF V600E status using US images. Other studies adopted machine learning methods to develop prediction models for BRAF V600E mutation based on the radiomics features extracted from US images in PTC. However, the AUC ranged from 0.65 to 0.73,^16^^,^^17^^,^^18^^,^^19^ indicating low to moderate performance, which was unsatisfactory for clinical application. Moreover, the studies above were limited by small sample size and lack of external validation, causing concerns about over-fitting and uncertain generalizability. In addition, a few studies focused on the association of BRAF V600E with novel US technologies, such as elasticity US^19^^,^^24^ and contrast-enhanced US.^25^ However, these technologies are neither widely available nor definitely recommended by current guidelines for thyroid cancer, which was the reason why we aimed at utilizing the grayscale US that is conducted as a clinical routine.

The BrafSwinT model established in this study showed an AUC of 0.911 and 0.869 for predicting the BRAF V600E mutation status in the validation and external test set, respectively. Its superior performance compared to previous studies was likely due to the advanced construction methods. BrafSwinT represents the pioneering application of state-of-the-art deep-learning models in the current clinical scenario. During the selection of the pre-trained backbone network, the SwinT model emerged as the optimal choice, which excelled in medical image processing by incorporating a shifted window-attention mechanism that enhanced local perceptibility. Subsequent modifications were made to the original SwinT model to optimize its performance specifically for the current task. In the preprocessing stage, the challenge of data category imbalance was addressed by employing oversampling techniques to augment the negative samples. Furthermore, contrast enhancement, flip, brightness enhancement, and random rotation were applied to bolster the overall quality and diversity of the data. In the modeling phase, Focal Loss was employed to enhance the model’s performance specifically for the small category (i.e., negative samples in this study). It also contributed to refining the model’s capacity to identify hard samples (i.e., images with measuring markers). In addition, the learning rate scheduling strategy of CosineAnnealingLR^26^ was implemented. This dynamic adjustment of the learning rate proved instrumental in accelerating training convergence and enhancing the generalization performance of the model. Besides, to enhance the feature input of the classifier, three clinical factors were integrated and concatenated with the image features. This delicate construction strategy attributed to the preferable performance of BrafSwinT compared to previous studies, demonstrating the intrinsic connection between US imaging and BRAF V600E mutation status in PTC. This connection was further corroborated by the focus on microcalcifications in US images both in the heatmaps of the current model and in a previous correlation study.^22^

The BrafSwinT model was well-prepared for clinical application with promising prediction efficacy in both the internal validation and independent external test set. The model utilized widely available grayscale US images collected by various US systems and multiple radiologists to simulate real-world clinical scenarios, enlarging its sphere of application. A relatively large cohort was investigated as the training set to enhance the model’s robustness. Additionally, the model generalization was scrupulously verified through a multi-center design.

### Limitations of the study

This study has several limitations. First, all the patients in the training and validation cohorts were collected from tertiary hospitals, so the applicability of the conclusions to PTC patients in other settings, such as primary care centers, requires further validation. Second, as a retrospective study, selection biases were not able to be fully avoided. Third, for patients with multiple PTC nodules, the BRAF V600E mutation results could not be attributed to any specific nodule, hence only the largest PTC nodule in each patient was analyzed. In addition, we made a great effort to address the challenge of data category imbalance, for example by employing oversampling techniques in the BRAF V600E negative group and using Focal Loss method in the modeling phase. However, the specificity and NPV of BrafSwinT were improved but still suboptimal in the external test set. Future studies with larger datasets and superior analysis methods are expected to further ameliorate the performance.

In conclusion, a BrafSwinT model was designed and tested using routinely acquired US and clinical data, showing promising predictive value in determining the BRAF V600E mutation status in PTC. The model could potentially be a useful tool as an alternative to conventional testing methods, thereby facilitating risk stratification and treatment decisions for the patients.

## Resource availability

### Lead contact

Further information and requests for model implementation should be directed to and will be fulfilled by the lead contact, Jianhua Zhou (zhoujh@sysucc.org.cn).

### Materials availability

This study did not generate new unique reagents.

### Data and code availability

• The deidentified data will be available upon reasonable request email to the corresponding author (zhoujh@sysucc.org.cn). The email should include the purpose and process of using the data, as well as the scope of personnel involved.
• The code for the model development is available online (https://github.com/JoSeunghyr/BrafSwinT). The website of the code is listed in the key resources table.
• Any additional information required to reanalyze the data reported in this work paper is available from the lead contact upon reasonable request.

## Acknowledgments

This study was funded by the 10.13039/501100001809National Natural Science Foundation of China (no. 82320108011).

## Author contributions

Conceptualization: Y.Y., C.Z., R.G., Y.Z., X.L., N.L., Y.L, X.H., X.T., R.M., C.P., J.Y., and J.Z. methodology: Y.Y., C.Z., J.Y., and J.Z. resources: Y.Y., R.G., Y.Z., X.L., N.L., Y.L, X.H., X.T., R.M., and C.P. software: Y.Y., C.Z., R.G., Y.Z., J.Y., and J.Z. writing – original draft: Y.Y. Writing – review and editing: C.Z., R.G., Y.Z., X.L., N.L., Y.L, X.H., X.T., R.M., C.P., J.Y., and J.Z. supervision: C.P., J.Y., and J.Z. funding acquisition: J.Z.

## Declaration of interests

The authors declare no competing interests.

## STAR★Methods

### Key resources table

**Table:** 

REAGENT or RESOURCE	SOURCE	IDENTIFIER
**Software and algorithms**		

R software	Version 4.0.2	https://www.r-project.org/
MedCalc	Version 19.0.4	https://www.medcalc.org/
Python	Version 3.7.13	https://www.python.org/
PyTorch	Version 1.8.0	https://pytorch.org/
Grad-CAM	Selvaraju et al.^27^	https://arxiv.org/abs/1610.02391
BrafSwinT	Code for this study	https://github.com/JoSeunghyr/BrafSwinT

### Experimental model and study participant details

#### Study population

This retrospective study was approved by the ethics committee of Sun Yat-Sen University Cancer Center (SYSUCC) with the written informed consent waived (B2023-185).

The patients were independently included in three cohorts: (1) the training cohort from SYSUCC, (2) the validation cohort from SYSUCC, and (3) the external test cohort from two other centers. Eligible patients in SYSUCC who underwent thyroid US and BRAF V600E testing from June 2017 to March 2023 were collected and randomized in a 9:1 ratio into the training cohort and the validation cohort. The validation cohort was not involved in the training process. The external test cohort consisted of eligible patients collected in the Gansu Provincial Cancer Hospital and the Fujian Provincial Cancer Hospital from May 2018 to July 2023. All participants in this study are Chinese.

The flowchart of patient selection is presented in Figure 1. In detail, patients satisfying the following criteria were included: (1) Patients with thyroid nodules pathologically diagnosed as thyroid carcinoma by fine needle aspiration or surgery. For patients with multiple thyroid carcinoma nodules, only the largest lesion was analyzed. (2) Patients with gray-scale US images of the targeted nodule. (3) Patients with available BRAF V600E testing results. The exclusion criteria were: (1) not PTC or having undetermined pathological results; (2) unmatched US and pathological results; (3) having undergone thyroid operation or radioactive iodine therapy before the US examination; (4) poor US image quality.

### Method details

#### US images and clinical parameters collection

US examinations were conducted by qualified radiologists as part of the routine clinical workflow in each institution, using multiple US systems equipped with linear array transducers with a frequency range from 8.4 to 18 MHz. The US systems used were listed in the Table S2. For each nodule, the most representative gray-scale images were acquired. The images were reviewed and collected by two qualified radiologists.

The sex and age of all the patients as well as the maximum size of the targeted thyroid nodules were recorded as clinical parameters. The BRAF V600E mutation status of the targeted PTC nodules was retrospectively obtained from the patients’ medical records.

#### Data preprocessing

The original US data were preprocessed before being input into the deep learning model. The region of interest (ROI) of each US image was manually annotated by two qualified radiologists with a bounding rectangle covering the nodule area and expanded by 3 mm in each of the four directions. This 3 mm expansion was adopted to reflect possible reactive changes surrounding the lesion as well as posterior echo. Subsequently, the ROI was cropped from the original US image based on the annotation. To mitigate overfitting, a data augmentation pipeline was applied during training, which included resizing, random cropping, random horizontal flipping, random brightness adjustments, and normalization.

#### Backbone network selection

Initially, the predictive capabilities of the pre-trained ResNeSt, ViT, and SwinT models in classifying the BRAF V600E mutation status in PTC nodules were examined in the training, validation, and external test set. The ResNeSt was trained for 110 epochs with an SGD optimizer, batch size of 15, learning rate of 1 × 10−3, weight decay of 0.05. Meanwhile, the ViT and SwinT were trained for 100 epochs with an SGD optimizer, batch size of 10, learning rate of 2 × 10−3, weight decay of 0.05. We used AUC and accuracy as criteria to evaluate the validation and testing performance of these models. The best-performing model was selected as the backbone network of the integrated model to be developed. Subsequent model development and evaluation processes were described in the results section with supportive data.

#### Heatmap generation

Heat maps were employed to illustrate the regions that the model focused on within the input images. Color coding was applied to pinpoint the regions of the image that the model deemed most informative. The Gradient-weighted Class Activation Mapping algorithm was utilized to generate these heat maps, which conveyed the significance of various spatial locations within the US images for predicting outcomes.

### Quantification and statistical analysis

#### Statistical analysis

Of the clinical parameters, the normally distributed continuous variables, non-normally distributed continuous variables, and categorical variables were compared using the two independent sample t-tests, Mann-Whitney U tests, chi-square tests or Fisher’s exact tests, respectively. Six metrics were employed to assess the classification performance of the deep-learning prediction models, comprising the AUC, accuracy, sensitivity, specificity, positive predictive value, and negative predictive value. The AUCs were compared using the two-sided DeLong test. A two-sided *p*-value of less than 0.05 was considered statistically significant. The statistical analysis was performed using R software version 4.0.2 (R Foundation for Statistical Computing) and MedCalc version 19.0.4 (MedCalc Software Ltd). The deep learning network was based on Python version 3.7.13 (Python Software Foundation) and PyTorch version 1.8.0 library (The Linux Foundation).
