# Supplementary material for: Deep learning model based on ultrasound images predicts BRAF V600E mutation in papillary thyroid carcinoma
Source: iScience. 2025 Apr 18;28(5):112482. doi: 10.1016/j.isci.2025.112482 (PMC12090294; doi:10.1016/j.isci.2025.112482)
Supplement: Document S1. Tables S1 and S2 [file mmc1.pdf]

iScience, Volume *28*

Supplemental information

Deep learning model based on ultrasound images predicts BRAF V600E mutation in papillary thyroid carcinoma

Yiwen Yu, Chengqian Zhao, Ruohan Guo, Yafang Zhang, Xiaoxian Li, Naxiang Liu, Yun Lu, Xu Han, Xiaofeng Tang, Rushuang Mao, Chuan Peng, Jinhua Yu, and Jianhua Zhou

Supplemental Information

		Actual BRAF V600E mutation status	
		Positive	Negative
Predicted BRAF V600E mutation status	Positive	98	6
	Negative	10	21

Table S2. The confusion matrix of the performance of BrafSwinT, related to Table 2. The table shows the actural and predicted BRAF V600E mutation status in the external test set.

Institute	Model	Manufacturer
Sun Yat-Sen University Cancer Center	LOGIQ E9, LOGIQ S8	GE Healthcare, Chicago, IL, USA
	Acuson Sequoia, Acuson Juniper	Siemens Medical Solutions USA, Malvern, PA, USA
	EPIQ 7	Philips Healthcare, Best, the Netherlands
	MyLab Twice	Esaote, Genoa, Italy
	Aloka Arietta 60	Hitachi Healthcare Corporation, Tokyo, Japan
	Aplio 400	Toshiba Medical Systems, Tochigi, Japan
	Resona 7T, I9S, DC-8	Mindray Medical International Co., Ltd., Shenzhen, China
Fujian Provincial Cancer Hospital	Acuson Sequoia	Siemens Medical Solutions USA, Malvern, PA, USA
	Aixplorer	SuperSonic Imagine, Aix-en-Provence, France
	iu22	Philips Healthcare, Best, the Netherlands
Gansu Provincial Cancer Hospital	LOGIQ E20, LOGIQ E9	GE Healthcare, Chicago, IL, USA
	EPIQ 7, EPIQ 5	Philips Healthcare, Best, the Netherlands
	Acuson X300	Siemens Medical Solutions USA, Malvern, PA, USA
	MyLab	Esaote, Genoa, Italy
	Resona 7T	Mindray Medical International Co., Ltd., Shenzhen, China
	S50	SonoScape Medical Co., Shenzhen, China

Table S2. A list of all the ultrasound machines used in the study, related to STAR Methods. The table shows all the ultrasound machines used in the three centers, as well as the models and manufacturers.
